# Acute Liver Failure due to Disseminated Varicella Zoster Infection

**DOI:** 10.1155/2018/1269340

**Published:** 2018-09-27

**Authors:** Elizabeth Caitlin Brewer, Leigh Hunter

**Affiliations:** Methodist Hospitals of Dallas, 1441 N Beckley Ave Dallas, TX 75203, USA

## Abstract

Acute liver failure (ALF) can be due to numerous causes and result in fatality or necessitate liver transplantation if left untreated. Possible etiologies of ALF include ischemia, venous obstruction, medications, toxins, autoimmune hepatitis, metabolic and infectious causes including hepatitis A-E, varicella-zoster virus (VZV), cytomegalovirus (CMV), herpes simplex virus (HSV), Epstein-Barr virus (EBV), and adenovirus with VZV being the most rarely reported. Pathognomonic skin lesions facilitate diagnosis of VZV hepatitis, but definitive diagnosis is secured with liver biopsy, tissue histopathology, culture, and specific VZV polymerase chain reaction (PCR). Antiviral treatment with intravenous acyclovir can be effective if initiated in a timely manner; however, comorbidities and complications frequently result in high mortality, especially in immunocompromised hosts as exemplified in this case presentation.

## 1. Introduction

Varicella zoster virus can cause two clinical syndromes, primary (chickenpox) and secondary (herpes zoster). Primary infection presents mainly in children with a generalized vesicular rash. The virus then establishes latency in the dorsal root ganglia and can later reactivate as “shingles” or herpes zoster, a localized dermatomal vesicular eruption. Cutaneous and extracutaneous dissemination can occur, most commonly in immunocompromised patients [1]. Fulminant hepatic failure due to VZV hepatitis is even more rare and deadly. In review of the literature, only 8 adult cases of acute liver failure from VZV were found, of which only 2 survived [2–9].

## 2. Case Presentation

**Figure 1:**
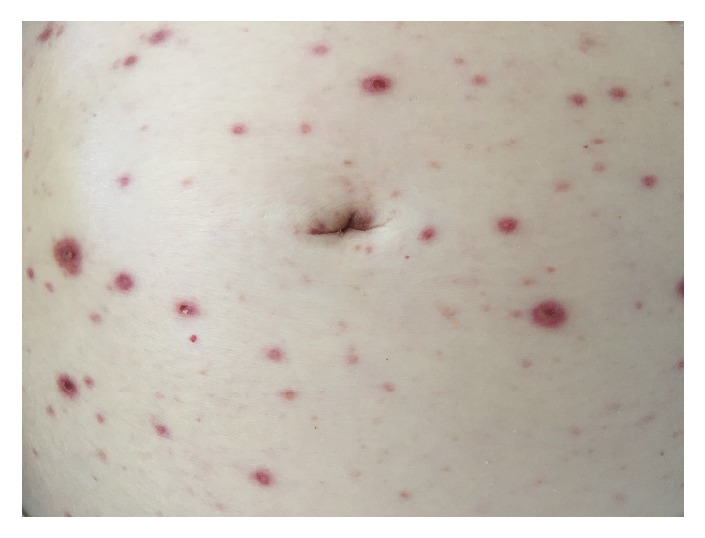
Maculopapular rash.

**Figure 2:**
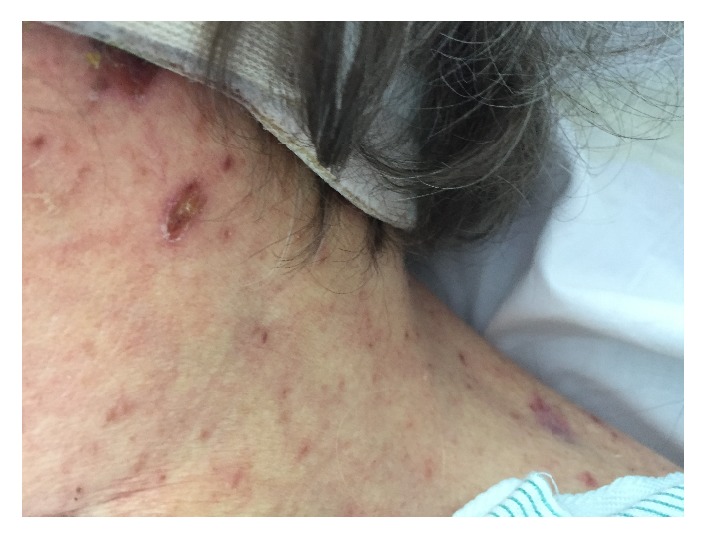
Crusted vesicle.

A 66-year-old Caucasian woman with past medical history of dermatomyositis, dysphagia, gastro-esophageal reflux, and hypertension presented to the emergency department (ED) with several days of mid-epigastric, constant, moderate intensity, nonradiating abdominal pain. Additionally, she reported 4-5 days of erythematous rash that began on her face and chest that then spread to her arms and abdomen (Figures 1-2). She also reported white “spots” in her mouth. At that time, CBC, CRP, ESR, CK, and UA were within normal limits. Lipase was 675 U/L and CMP was remarkable for sodium 129 mEq/L, amino alanine transferase (ALT) 158 U/L, and aspartate aminotransferase (AST) 111 U/L; the rest of the CMP including alkaline phosphatase (ALP) was normal. CXR was normal and abdominal radiograph showed evidence of constipation. An abdominal ultrasound was ordered due to elevated lipase and LFTs and showed no evidence of gallbladder dysfunction or liver lesions. She was diagnosed with pancreatitis, thrush, and folliculitis and was discharged home with clear liquid diet orders and prescriptions for nystatin oral solution and oral doxycycline for possible secondary skin infection. Two days later, she returned to the ED with persistent symptoms and decreased urine output. She reported nausea, constipation, and worsened dysphagia, but denied vomiting, weight change, night sweats, fever, chills, chest pain, cough, and shortness of breath. She also denied pertinent past surgeries, family history, recent travel, sexual activity, drug use, and alcohol and tobacco use. She reported allergy to penicillin. Her medication list included prednisone, mycophenolate mofetil (which she held since previous ED visit per doctor recommendations), trimethoprim/sulfamethoxazole (T/S), nystatin oral suspension, carvedilol, ranitidine, estradiol, calcium, and vitamin D. She was told by her dermatologist not to fill the doxycycline prescription from the ED and increase the dose of T/S.

**Figure 3:**
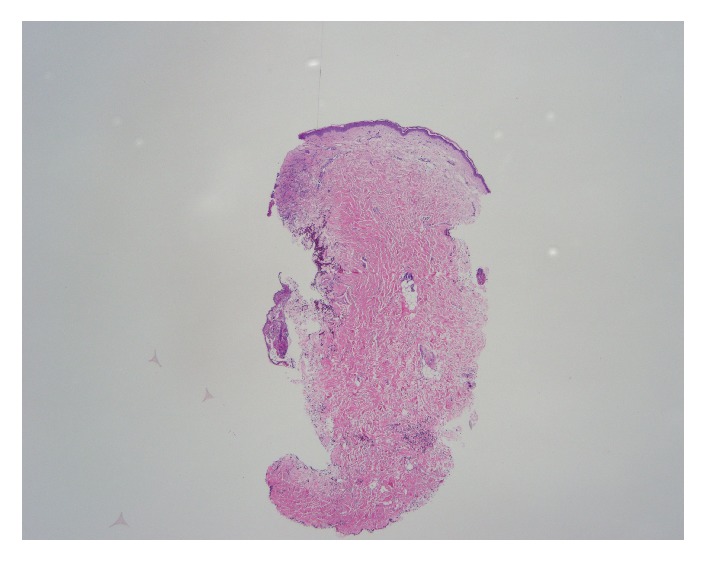
Skin biopsy.

**Figure 4:**
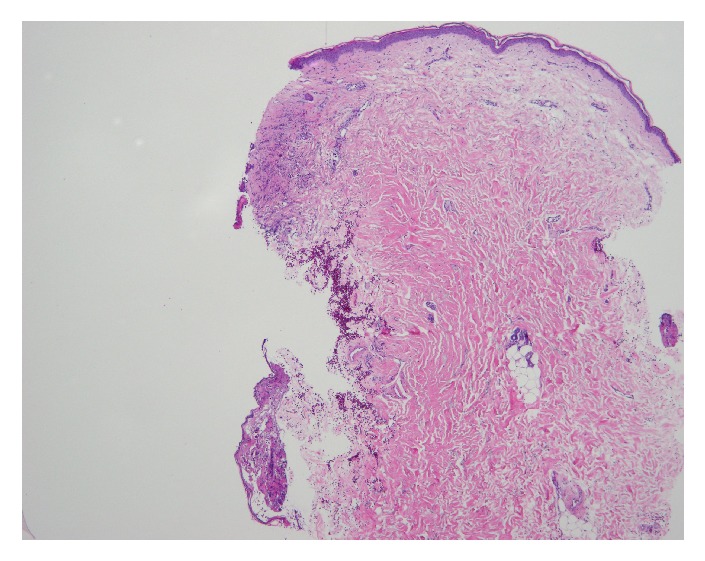
Skin biopsy: intact epidermis on one side and lesion on the other.

**Figure 5:**
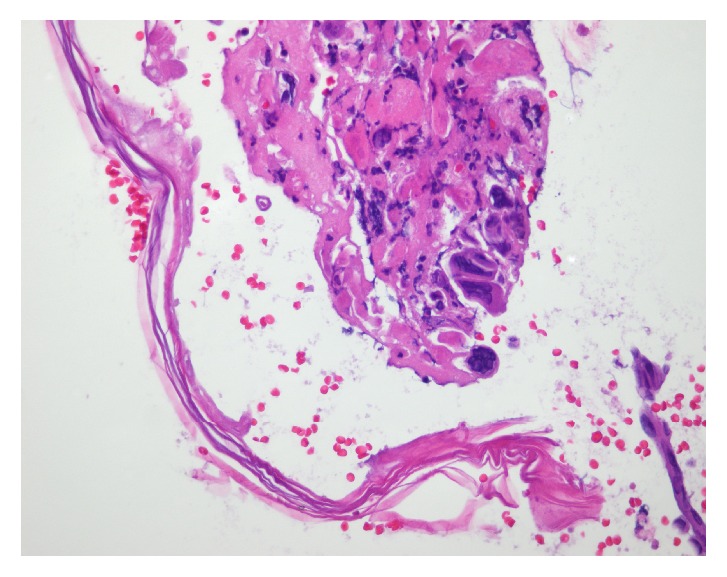
Skin biopsy: viral cytoplasmic effect including multinucleated cells and marginalization of chromatin.

On physical examination, the patient was alert and oriented with normal vital signs. The exam was significant for oral thrush, but normal heart, lung, and abdominal exams. Skin exam showed a diffuse maculopapular eruption with a few vesicles on face, trunk, and extremities. Significant laboratory data was as follows: AST 1389 U/L, ALT 1570 U/L, ALP 68 U/L, international normalized ratio (INR) 1.6, and prothrombin time (PT) 18 seconds. Complete abdominal ultrasound demonstrated normal gallbladder without stones, no biliary ductal dilation, no focal liver lesions, and no ascites or abnormal fluid collections. The patient's dermatologist had performed skin biopsies 2 days prior to admission that showed multinucleated giant cells with viral inclusions suggestive of some type of herpes virus infection (Figures 3–5). The patient was initiated on intravenous (IV) acyclovir, micafungin, vancomycin, aztreonam, and stress dose steroids for presumed disseminated herpes simplex with possible secondary bacterial infection and sepsis. Over the subsequent 48-72 hours, AST and ALT increased to the 4000s, INR increased to 1.8 and PT to 20.6. Due to worsening acute liver failure, she was transferred to our facility for liver transplant evaluation.

**Figure 6:**
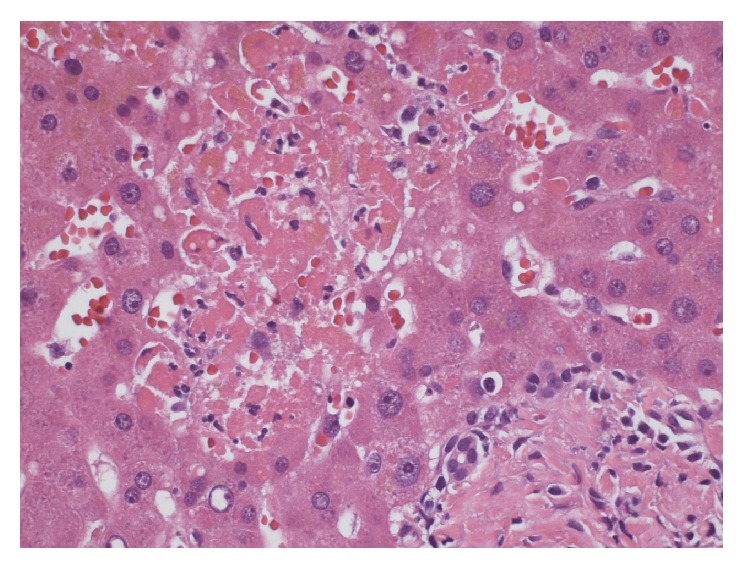
Hepatocytes with frank necrosis.

**Figure 7:**
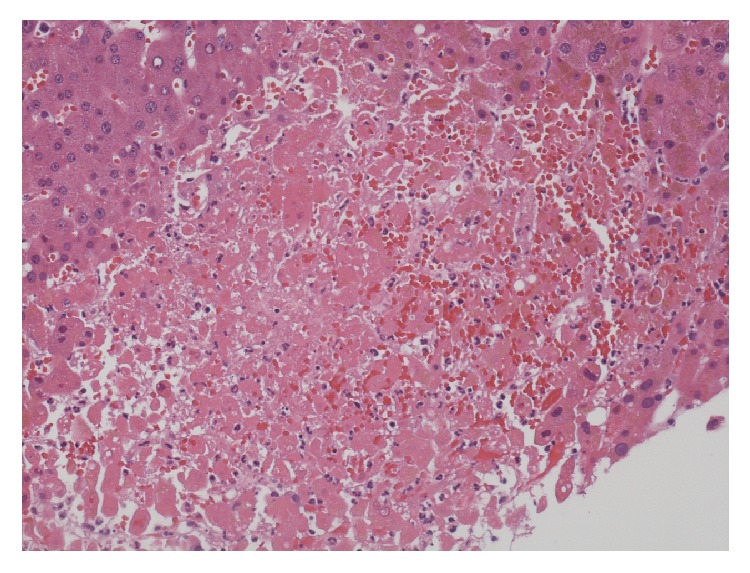
Larger foci of hepatocytes with frank necrosis.

**Figure 8:**
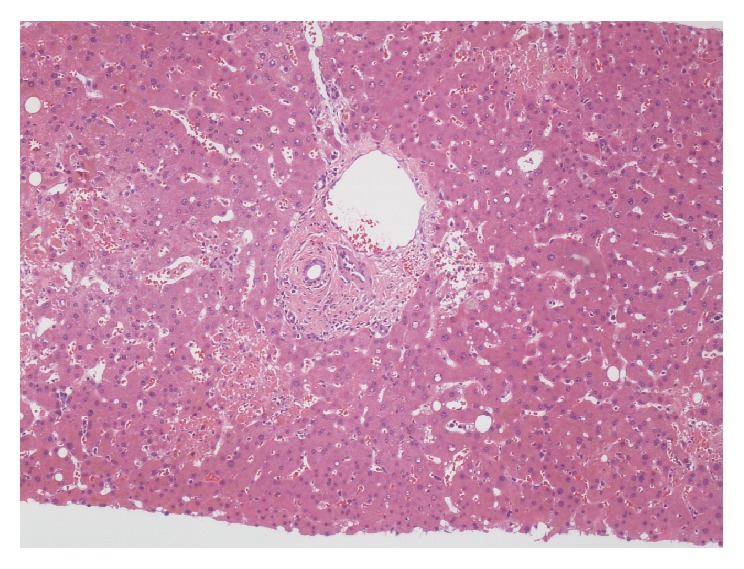
Areas of necrosis abutting portal triad with chronic minimal inflammation; no significant steatosis seen and no periportal fibrosis or vasculitis appreciated.

On arrival to our hospital, her skin lesions were thought to be most consistent with VZV and skin biopsy cultures from the outpatient dermatologist later confirmed VZV. IV acyclovir and antibiotics for secondary bacterial sepsis were continued. As part of her liver transplant evaluation, extensive serologic investigation ensued. Acute hepatitis A-E serologies, ANA, IgG4, smooth muscle antibody (Ab), LKM-1 Ab, ceruloplasmin, a-1 antitrypsin, mitochondrial M2 Ab, AFP, HIV, HSV and EBV PCRs, blood cultures, galactomannan, and cryptococcal antigen were submitted and later found to be negative. She was also found to be pANCA MPO positive and PR3 negative. Liver biopsy was performed which revealed multiple areas of necrotic hepatocytes (up to 35% of liver parenchyma) (Figures 6-7) in zones 2 and 3 of the liver. This was associated with some bile extravasation and acute inflammation. No signs of bridging or confluent necrosis were seen. A trichrome stain outlined regions of immature deposition of collagen near necrotic areas. This stain also showed increased perivenular fibrosis around the central veins but no evidence of periportal fibrosis. The portal triads showed nominal chronic inflammation with small lymphocytes, rare large lymphocytes, and a few scattered neutrophils. There was no bile duct injury, paucity, or vasculitis. The hepatocytes did not demonstrate significant steatosis, but there was bile stasis in a few canalicular spaces and hepatocytes. Viral inclusions were not seen. An iron stain showed no accumulation of hemosiderin within the hepatocytes. PCR was negative for HSV, EBV, and CMV and positive for VZV. The necrosis also abutted portal triads seen in the specimens (Figure 8). A Periodic acid-Schiff stain with diastase did not show cytoplasmic globules. Blood PCRs were positive with high levels of VZV and low levels of CMV. The CMV viremia was attributed to secondary reactivation due to her severely immunosuppressed state. VZV immune globulin was considered as therapy, but IVIG was administered instead due to her severe coagulopathy and thrombocytopenia. Outpatient skin biopsy cultures later confirmed VZV; PCR was positive for VZV and negative for HSV.

Her hospital stay was complicated by multidrug-resistant Enterobacter cloacae hospital acquired pneumonia and bacteremia, respiratory failure requiring prolonged intubation, and multiple organ failure. VZV PCR copies decreased with treatment, but her severity of illness and active infection prevented liver transplantation. The patient's code status was eventually changed to “do not resuscitate” and she expired.

## 3. Discussion

Reactivation of VZV as “shingles” is a common occurrence, but acute liver failure (ALF) due to VZV is exceedingly rare with high mortality [2–9]. The differential diagnosis of ALF includes multiple etiologies including ischemia, venous obstruction, medications, toxins, autoimmune hepatitis, and metabolic and infectious etiologies (predominantly viruses including hepatitis A-E, VZV, CMV, HSV, EBV, and adenovirus) with VZV being the one most rarely reported. By imaging and history, the patient was less likely to have venous obstruction or ischemia from an unrecognized hypotensive event that triggered her hepatitis. She denied exposure to toxins, but had been on prophylactic trimethoprim/sulfamethoxazole for long duration; this was a consideration for drug induced liver injury, but was refuted by biopsy, PCR, and culture results. There was no history of ingestion of other toxins known to cause acute hepatitis before onset of illness. Likewise, the AST to ALT ratio was not in the classic pattern (2:1) for alcoholic hepatitis.

In the setting of VZV hepatitis, definitive diagnosis is made by liver biopsy, histopathology, culture, and VZV PCR. Of note, some cases have been shown to be pANCA positive as well. Thus it is important to utilize physical examination clues to prompt ordering of proper tests along with early empirical antiviral therapy.

Disseminated varicella zoster is most common in immunocompromised patients [1]. Resultant fulminant liver failure from VZV is even more rare and deadly. In review of the literature, only 8 adult cases of acute liver failure from this organism were found, of which only 2 survived. These cases are summarized in Tables 1 and 2. One case report from France involved a 35-year-old woman from the Ivory Coast with past medical history (PMH) of HIV, HBV, and recent neurotoxoplasmosis [2]. A second case report from Spain was a 43-year-old male heart transplant recipient 9 months prior to the time of ALF from a VZV episode [3]. In these cases, IV acyclovir was the staple of treatment [2, 3]. The heart transplant patient also was treated with VZV immune globulin and emergent liver transplant [3]. Other reported cases that did not survive included a 49-year-old German man with PMH of alcohol and tobacco abuse 15 days post radical neck dissection and laryngectomy for laryngeal squamous cell carcinoma who presented for abdominal pain. He received supportive care and was diagnosed post mortem [4]. A Japanese 47-year-old man with multiple myeloma status post chemotherapy, corticosteroids, 2 stem cell transplants, moderate graft versus host disease, and relapse of the myeloma necessitating additional chemotherapy and corticosteroids presented with generalized fatigue. He was treated with fresh frozen plasma, platelet transfusions and was also diagnosed post mortem [5]. Another case reported from Italy was a 49-year-old man with no PMH except treatment for pharyngotonsillitis with steroids and antibiotics 15 days before presentation with retrosternal chest pain and truncal rash. He was treated with IV acyclovir, VZV immune globulin, and total hepatectomy, but was unable to receive a donor liver in time [6]. The next case was of a 15-year-old Roman male with no PMH who was treated with IV acyclovir, MARS (molecular adsorbent re-circulating system), and blood product transfusions. Diagnosis was confirmed with a post mortem liver analysis [7]. An additional case was of a 26-year-old Czech female with diagnosis of multiple sclerosis 3 months prior to admission followed by treatment with steroids who presented with abdominal pain and vomiting and later developed a generalized rash. She was originally treated with oral acyclovir, but because of progressive worsening was changed to IV acyclovir. Despite aggressive treatment, she also expired [8]. The final reported case involved a 64-year-old Caucasian woman in Vermont who was 14 months post esophagogastrectomy/splenectomy and came to the hospital with headache, malaise, and fever. She was diagnosed post mortem with VZV by acute and convalescent antibody titers and liver analysis. She was treated supportively with vitamin K but subsequently died as well [9].

As shown, early IV acyclovir is key to treatment of VZV acute liver failure. Other considered therapies include VZV immune globulin, liver transplant, IVIG, and supportive care [2–9]. Since this cause of liver failure has such high mortality rates and early treatment is critical to survival, VZV hepatitis should be considered in the differential diagnosis of all patients with liver failure who present with a rash.

## Figures and Tables

**Table 1:** Case reports: survivors [2, 3].

**Pt Info**	**PMH**	**Symptoms**	**Treatment**	**Diagnosis**	**Liver Biopsy**
35 y/o African F from Ivory Coast	HIV, HBV and recent neurotoxoplasmosis	Chest pain	IV acyclovir	Vesicle swab VZV + by direct IF and culture; liver bx	>50% hepatic necrosis and inclusion bodies

43 y/o M	S/p heart transplant 9 months earlier	N/V, epigastric pain	IV acyclovir, VZV immune globulin, emergent liver transplant	Skin lesion biopsy: HSV – VZV + liver biopsy	Transjugular liver bx: signs of herpetic hepatitis; histology of hepatectomy: hepatic necrosis consistent with VZV infection

**Table 2:** Case reports: nonsurvivors [4–9].

**Pt Info**	**PMH**	**Symptoms**	**Treatment**	**Diagnosis**	**Liver Biopsy**
49 y/o M	ETOH and tobacco abuse, 15 days post radical dissection neck and laryngectomy for SCC larynx	Abdominal pain, fever, restlessness	“Intensive supportive care”	Post mortem via liver analysis	Post mortem: liver VZV DNA +, hepatic necrosis with intranuclear inclusion bodies

47 y/o Japanese M	MM s/p chemo, steroids, 2 stem cell transplants, moderate GVHD and relapse of MM with more chemo and steroids	Generalized fatigue	FFP, platelets	Retrospective VZV PCR + blood and liver analysis	Autopsy: + anti-VZV IgG stain of liver with hepatic necrosis seen

49 y/o M	No PMH except treatment for pharyngotonsillitis 15 days prior with abx and prednisone	Acute retrosternal pain	IV acyclovir, VZV immune globulin, total hepatectomy	Skin cytology c/w herpes family virus & immuno-cytochemistry stain VZV +; blood VZV DNA +	Liver bx: necrosis only; Post mortem liver VZV DNA +

15 y/o M	None	Fever, abdominal pain, myalgia, skin vesicles	IV acyclovir, MARs	Post mortem liver analysis	Post mortem liver analysis: hepatic necrosis, multinucleation and intranuclear inclusions of Cowdry A bodies; liver VZV PCR +

26 y/o CF	Diagnosed with MS 3 months prior and treated with steroids	Abd pain and vomiting	PO acyclovir → IV acyclovir	Blood and urine VZV PCR +; post mortem liver analysis	Post mortem liver: hemorrhagic necrosis and VZV PCR +

64 y/o CF	14 months post-op esophago-gastrectomy & splenectomy	Fever, malaise, HA	Vit K	VZV titers D4: 1-64 → D7: 1-256; liver autopsy analysis	Autopsy liver: hemorrhagic necrosis and signs herpes family virus including Cowdry A intranuclear bodies; EM: intracellular virions consistent with herpes family virus

## Additional Points

*Learning Points/Take-Home Messages*. (1) VZV should be considered as a cause of acute liver failure in the proper clinical setting. (2) Diagnosis of VZV is dependent on liver biopsy, histopathology, culture of tissue, and PCR. (3) Early antiviral medication is essential to decrease morbidity and mortality from disseminated VZV infection. (4) Disseminated VZV with ALF has a very high mortality rate.
